# Institutional risk factors for norovirus outbreaks in Hong Kong elderly homes: a retrospective cohort study

**DOI:** 10.1186/1471-2458-11-297

**Published:** 2011-05-11

**Authors:** Hualiang Lin, Sammy Ng, Shelley Chan, Wai Man Chan, Krystal CK Lee, Suzanne C Ho, Linwei Tian

**Affiliations:** 1 School of Public Health and Primary Care, Chinese University of Hong Kong, Hong Kong SAR, China; 2 Department of Health, Hong Kong SAR, China

## Abstract

**Background:**

Most of the institutional outbreaks of norovirus in Hong Kong occur in elderly homes, the proportion being 69% in 2006. Residents in elderly homes are a special population seriously affected by norovirus infections, it is necessary to investigate the risk factors of the norovirus outbreaks in Hong Kong elderly homes at the facility level.

**Methods:**

A cohort of 748 elderly homes was followed up from January 2005 to December 2007; each elderly home was treated as one observation unit and the outcome event was the norovirus outbreak. Cox regression models were fitted to estimate the rate ratio (RR) and 95% confidence interval (CI) for the potential risk factors.

**Results:**

A total of 276 norovirus outbreaks were confirmed during the study period; the outbreak rate was 12.2 (95% CI: 9.9-14.6) per 100 home-years; elderly homes with a larger capacity (RR = 1.4, 95% CI: 1.3-1.5 (per 30-resident increment)), a higher staff-to-resident ratio (RR = 1.2, 95% CI: 1.1-1.3 (per 1/30 increment) and better wheelchair accessibility (RR = 2.0, 95% CI: 1.3-3.2) were found to have an elevated norovirus outbreak rate in Hong Kong elderly homes; Elderly homes with partitions between beds had a lower rate of norovirus outbreaks (RR = 0.6, 95% CI: 0.4-0.8).

**Conclusions:**

Elderly home capacity, staff-to-resident ratio and wheelchair accessibility were risk factors for norovirus outbreaks in Hong Kong elderly homes. Partitions between beds were a protective factor of norovirus outbreaks. These results should be considered in the infection control in Hong Kong elderly homes.

## Background

Hong Kong is facing an ageing population. In 2006, 12.4% of Hong Kong population were over 65 years old [1]. The population projection showed that the proportion of the elderly would go up to 21.9% in 2026 and 26.8% in 2033, respectively [2]. In 2005, there were about 60,000 elderly people residing in elderly homes, accounting for about 7% of the population aged 65 years old and above [3]. Residential care homes for the elderly are an important part of the health care system for the old age group. Evidence has shown that infection is an important cause of morbidity and mortality in the institutionalized elderly [4]. An atypically high level of norovirus outbreak has been noticed in Hong Kong in early May 2006; most of the outbreaks were located in the elderly homes [5]. Healthcare-associated norovirus outbreaks are an increasingly recognized problem; better understanding of the epidemiology of these events is needed [6-8].

Norovirus is a group of closely related and highly infectious viruses which was first reported following an outbreak of gastroenteritis in Norwalk, Ohio in 1972 [9]. In recent decades, Norovirus outbreaks have presented as a growing challenge in health care facilities in many countries worldwide [10-13]. In Hong Kong, norovirus has been recognized as a major cause of both sporadic cases and outbreaks of acute gastroenteritis [14-17]. In the world, norovirus has been identified as the cause of 73% to more than 95% of non-bacterial gastroenteritis outbreaks and approximately half of all gastroenteritis outbreaks [18]. Norovirus is extremely infectious, and as few as 10 to 100 particles may be enough to initiate an infection. These viruses are also highly resistant to inactivation by freezing, heating to 60°C, and treatment with ether, ethanol, or detergent-based cleaners. These characteristics make norovirus a major public health concern [19].

Norovirus are notoriously difficult to control in health care settings. They can affect all age groups (including both residents and staff) and an infection can be caused with a very small dose [20]. The viruses have several transmission modes, including food-borne transmission and person-to-person transmission by direct and indirect contact with vomitus or faeces from infected persons or contaminated objects. Vomitus-oral transmission may be particularly important in health care environment. There is frequently no prodrome to norovirus gastroenteritis, so an infected person may project vomit; the resulting aerosol of virus particles can be swallowed inadvertently or settle and contaminate environments [8,21]. Norovirus can persist in the environment for a long time, even more than 3-4 weeks, combined with its high transmissibility and high resistance to disinfectants, likely contributed to the large number of norovirus outbreaks [22]. Systematic evaluation of the institutional risk factors (staffing support, resident characteristics and environment and facilities) for norovirus outbreaks in Hong Kong elderly homes can help the design of prevention measures for norovirus outbreaks in these homes.

Most of the institutional outbreaks of norovirus in Hong Kong occurred in elderly homes, the proportion being 69% (182 out of the total 265 episodes of outbreak) in 2006 [23,24]. Residents in elderly homes are a special population seriously affected by norovirus infections. In the nursing homes worldwide, norovirus is the most common cause of gastroenteritis and several such outbreaks have resulted in deaths due to aspiration or to exacerbations of other chronic diseases [25]. Indeed, this mild self-limited infection can lead to significant morbidity and mortality in residents of elderly homes [26].

The current study was to examine the institutional factors that may predispose to norovirus outbreaks in Hong Kong elderly homes using a retrospective cohort study, which can help the formulation of future guidelines of norovirus infection control and prevention measures at the facility level.

## Methods

The cohort consisted of 748 elderly homes in Hong Kong. The baseline information on the potential predictor variables of each elderly home, in terms of resident characteristics, staffing support and environment and facility conditions, had been collected by the Territory-wide Infection Control Checklist Survey in 2004, and followed up annually during 2005-2007; the survey information included home capacity (number of residents in each elderly home), percentage of residents older than 75 years old, number of nursing staff, visits by Hospital Authority Community Geriatric Assessment Team (CGAT), visits by medical officers, supply of partitions between beds, air conditioner supply, wheelchair accessibility, supply of special isolation area for infection, hygiene condition of toilets and kitchens, staff infection control training. Wheelchair accessibility was defined as whether the wheelchairs were allowed to go to other rooms within the elderly home; the hygiene condition of toilets and kitchens was assessed based on the evaluation of the survey conducted by the Elderly Health Service (EHS) of the Department of Health (DH).

In Hong Kong, the elderly homes are regulated by the Social Welfare Department (SWD) under the Residential Care Homes (Elderly Persons) Ordinance. Elderly homes in Hong Kong are run by both the private sectors and non-governmental organizations, majority of which are operating subsidized services with government subvention. According to the Codes of Practice for Residential Care Homes, all elderly homes are requested to designate an infection control officer for infectious disease prevention and control, and the outbreaks reporting in the elderly homes is mandatory, the home managers and the infection control officers are requested to report any suspected infectious disease outbreak within the elderly homes [27].

Information on the norovirus outbreaks occurring among the elderly homes during the follow-up period was extracted from the Public Health Information System (PHIS) of DH. In general, Hong Kong Depart of Health received notification of suspected outbreaks from various sources, e.g. clinicians of the hospital (if some victims were admitted to the hospital) or the staffs of the elderly homes. There is a standard protocol for outbreak management and field sampling procedures. When the notification was received, DH would conduct investigation by collecting epidemiological information from the victims, their relatives and staff of the elderly homes, and at the same time, stool samples were collected within 48 hours of symptom onset from all patients when initially observed and processed immediately for RNA extraction; diagnosis of norovirus infection and its quantitation were based on real-time reverse transcription-PCR assay of stool samples as described elsewhere [28]; and inspection and control measures would be implemented as well; the surveillance would only be ended if there was no more new case for 6 days from the last case. When the surveillance was over, the information would be summarized and recorded in the PHIS system, including details on first date of outbreak onset, last date of outbreak, number of persons infected, maximum, minimum and mean age of infected persons and the laboratory test result of each episode. The protocol of the laboratory test has been described elsewhere [5,29]. Briefly, stool specimen, food remnant, or food samples were collected during the surveillance, and tested by RT-PCR. In the current study, the "outbreak" was defined as the episode with at least two cases from a same elderly home presenting symptoms of acute onset of diarrhoea with or without vomiting in the absence of a common food source and other common symptoms include abdominal pain and fever. It is a "laboratory-confirmed norovirus outbreak" when at least one specimen was norovirus positive by laboratory testing, which was considered as an event in the current study. Homes that were closed during the follow-up were treated as censored unless there was an outbreak before the closure. Approval to conduct this study was granted by the relevant ethics committees of the Chinese University of Hong Kong (Shatin, New Territories, Hong Kong Special Administrative Region, China).

**Figure 1:**
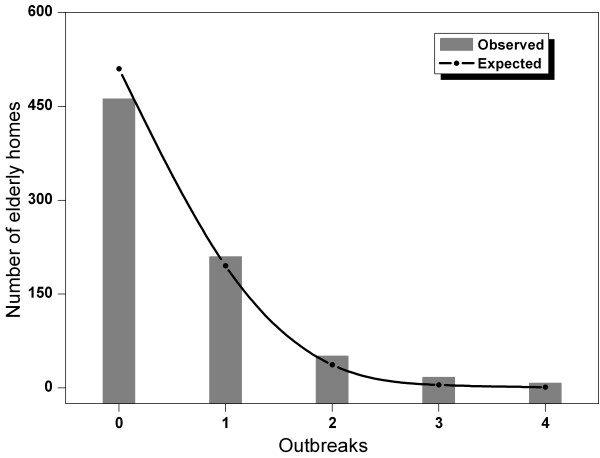
**Distribution of observed norovirus outbreaks in Hong Kong elderly homes and expected Poisson distribution (based on rate of 12.2 outbreaks per hundred home-years)**. Bar: observed; line: expected.

In cohort studies, subjects (elderly homes in this study) might have multiple outcome events (norovirus outbreaks in this study), which could not be assumed to be independent. The method of Wei et al. was used in this study; this method accounted for clustering in multiple failure data using robust estimates for the standard error of Cox proportional hazards [30]. However, the likelihood ratio test (LRT), used in assessing a variable's significance in multivariable models, may be invalid when standard errors are calculated by the robust method. For this reason, we examined the distribution of observed norovirus outbreaks to check clustering compared with the expected Poisson distribution, based on the observed outbreak rate (Figure 1). Outbreaks appeared to follow Poisson distribution, which lent weight to the nonindependence assumption of the norovirus outbreaks among the elderly homes.

In the present study, each elderly home was treated as one observation unit and the outcome event in the Cox model was the norovirus outbreak status. The survival time was determined as the number of days from January 1, 2005 to the onset of an outbreak or the end of the study (December 31, 2007). The independent variables in the Cox regression analysis included characteristics of the elderly homes and residents, staff supporting; such as home capacity (number of residents in one home), staff-to-resident ratio, percentage of the residents older than 75 years old, percentage of bedridden residents, infection control training, visits by CGAT or medical officers, supply of isolation area and air conditioners, hygiene condition of toilets and kitchens, wheelchair accessibility, partition between beds, etc., of which the former four variables were treated as continuous variables, all others were taken as categorical variables. In the current study, some characteristics of the elderly homes might change over the follow-up period, that is, the values of some variables were not fixed over time; for example, one elder home might have 50 residents in 2005; then the number increased to 60 in 2006; and then decreased to 40 in 2007; these variables were treated as time-varying variables in the Cox regression models [31]. Univariate analysis was performed for each variable first. Variables that had a p value of less than 0.10 in the univariate analysis were included in the multivariate Cox regression models. If two variables were believed to be proxies for similar biologic meaning, and were highly correlated with each other, the one most significant in the univariate analysis was included in the multivariate model.

One of the main assumptions of the Cox proportional hazard model is proportionality. We checked proportionality by including time-dependent covariates in the model. Time dependent covariates are interactions of the predictors with time; in this analysis we chose to use the interactions of the finally remained variables with log(time) because this is the most common function of time used in time-dependent covariates [32]. The statistical analysis was also carried out to include the eleven "self-care hostels" in order to explore whether this exclusion has presented any potential bias. All data management and analyses were performed using SAS 9.1 (SAS Institute, Inc., Cary, North Carolina).

## Results

A total of 760 elderly homes were in operation in Hong Kong in 2004, one of which was excluded because of duplicate ID number; another eleven homes, named "self-care hostels" were excluded because they were not quite comparable to other homes in terms of the residents' health profiles, living status, etc.; all the remaining 748 elderly homes accommodating 57,321 elderly residents were included in the final analysis.

During the study period, 276 laboratory confirmed norovirus outbreak episodes occurred in Hong Kong elderly homes, giving an overall outbreak rate of 12.2 episodes per hundred home-years (95% CI: 9.9-14.6) or 0.004 episodes per thousand bed-days (95% CI: 0.003-0.005). In total, 3,452 residents were infected; to ignore the outbreaks that only one resident was infected, the overall incidence was 2.0 per hundred person years (95% CI: 1.9-2.1).

### Univariate analysis

Table 1 illustrated the univariate analysis results. Norovirus outbreak rates were found to differ: homes with larger capacity, higher staff-to-resident ratio, higher fraction of older residents and better wheelchair accessibility were found to have an elevated norovirus outbreak rate; while supply of partitions between beds was found to be related to decreased rates. Percentage of bedridden residents was found to be marginally related to rates of outbreak.

**Tables 1:** Crude RRs and 95% Cls for potential risk factors related to norovirus outbreaks in Hong Kong elderly homes

Factors	Home-years	Outbreaks	RR	95% CI	P value
Home capacity (per 30-resident increment)			1.5	1.4-1.6	<0.0001
Staff-to-resident ratio (per 1/30 increment)			1.2	1.1-1.3	<0.0001
% residents older than 75 yrs (per additional 10%)			1.2	1.1-1.3	0.0003
% bedridden residents (per additional 10%)			0.9	0.8-1.0	0.07
Infection control training					
No	1024	1	1		
Yes	8259	275	3.5	0.5-25.6	0.2
Visits by CGAT					
No	15302	41	1		
Yes	6831	235	1.3	0.9-2.0	0.2
Visits by medical officers					
No	267	14	1		
Yes	8094	262	0.6	0.3-1.1	0.1
Isolation area					
No	2865	101	1		
Yes	5496	175	0.9	0.7-1.2	0.5
Air conditioner supply					
No	157	12	1		
Yes	8204	264	0.4	0.2-1.1	0.1
Hygiene condition of toilets					
High	113	2	1		
Low	8248	274	1.9	0.5-7.4	0.3
Hygiene condition of kitchens					
High	438	13	1		
Low	7923	263	1.1	0.7-1.9	0.7
Wheelchair accessibility					
No	1794	21	1		
Yes	6567	255	3.5	2.2-5.6	<0.0001
Partition between beds					
No	2111	151	1		
Yes	6251	125	0.25	0.19-0.34	<0.0001

No difference was found in term of staff infection control training, visits by Hospital Authority Community Geriatric Assessment Team (CGAT), visits by visiting medical officers, whether there was an isolation area for infections, whether there was air conditioner supply and the hygiene of toilets and kitchens as shown in Table 1.

### Multivariate model

Significant factors identified in the univariate analysis (P < 0.10) were included in the multivariate Cox regression model to further explore their relationships with the norovirus outbreak rates. The proportion of bedridden residents and proportion of residents older than 75 years old were believed to have similar biological meaning, in the multivariate model, proportion of residents older than 75 years old was included.

Table 2 showed the results of the multivariate model. Home capacity and staff-to-resident ratio were statistically related to elevated rates of norovirus outbreaks. In the multivariate analysis, better wheelchair accessibility was still significantly related to increased norovirus outbreak rates after adjusting for potential confounding factors (RR = 2.0, 95% CI: 1.3-3.2); while supply of partitions between beds was still a protective factor (RR = 0.6, 95% CI: 0.4-0.8). And percentage of residents older than 75 years was not statistically significant after adjusting for potential confounding factors. When the eleven "self-care hostels" were included in the statistical analysis, the results remained similar.

**Table 2:** Multivariate analysis of potential risk factors related to norovirus outbreaks in Hong Kong elderly homes

Factors	RR	95% CI	P value
Home capacity (per 30-resident increment)	1.4	1.3-1.5	<0.0001
Staff-to-resident ratio (per 1/30 increment)	1.2	1.1-1.3	<0.0001
% residents older than 75 (per additional 10%)	2.1	0.7-6.1	0.2
Wheelchair accessibility			
No	1.0		
Yes	2.0	1.3-3.2	0.002
Partition between beds			
No	1.0		
Yes	0.6	0.4-0.8	0.002

The proportionality assumption tests for four variables were not significant either individually or collectively, so we did not have enough evidence to reject proportionality and assumed that we have satisfied the assumption of proportionality for this model.

## Discussion

The current study, with an analytical epidemiology study design, has produced unique insights into the potential risk factors of norovirus outbreaks in Hong Kong elderly homes. The finding that larger elderly homes had higher risk of norovirus outbreaks was consistent with the findings of one prospective cohort study in England and one case-cohort study in New York [33,34]. The higher risk in larger elderly homes might be due to the possible difference in infection control practice, resulting in more outbreaks being discerned and reported by larger elderly homes. However, when factors indicating the infection control practices, such as isolation areas for infection, staff infection control training and hygiene of toilets and kitchens were controlled for in the multivariate Cox regression model, it was still highly associated with increased norovirus outbreaks. Thus home capacity itself might lead to a true increase in the outbreaks of norovirus in Hong Kong elderly homes. Homes with more residents had a higher probability of introducing pathogens from the community as well as transmission within the homes. The relative difficulty of serving more residents within one elderly home, as well as the increased person-to-person contact among residents, their relatives, visitors and staff with different characteristics, might create higher chance of introduction and transmission of norovirus in larger homes than in smaller ones [34].

The relationship between nurse staffing and health care quality has been extensively examined in the past decades [35-43]. However, the effect of nurse staffing on the infection outbreaks in health care facilities has not been conclusive. Some previous studies [33,38,43-47] found that nurse understaffing was one potential risk factor for nosocomial infection risks; inconsistent with the previous studies, the current study found that homes with a higher staff-to-resident ratio had an elevated risk of norovirus outbreaks. A similar relationship was also observed in a study of respiratory infection in Hong Kong elderly homes by a previous analysis, which found that homes with nurse support had higher risk of respiratory infections [48]. These findings might have also suggested another possible norovirus transmission mode, "attendant-borne transmission" [49]. While tending to patients, the health care staff might inadvertently carry pathogens from one resident to another; they may do this by direct contact through their hands or indirectly through their use of health care facilities. Such attendant-borne transmission has been reported as the major route for some hospital-acquired pathogens [50], and might be the case in the current study, as in the 276 outbreaks, health care staff were infected in about half (132/276) and low hand washing compliance has been reported in Hong Kong health care workers [51].

The higher risk among homes with higher staff-to-resident ratio might be explained in part by the fact that pathogens could be introduced by the nursing staff from outside community. The nursing staff usually has lower probability to be infected, partly because they are less vulnerable than the older population [5], and they may have generated some immunity to the organisms. The wide activity range of the nursing staff in the elderly homes enabled them to serve as a transmission vector among the residents, and elderly homes and outside community, as reported in one hospital outbreak, norvirus was detected in the asymptomatic staff [52].

Elderly homes with better wheelchair accessibility had elevated risk of norovirus outbreaks. The underlying mechanism remains unknown. Increased mobility of the residents may result in higher chance of norovirus transmission within the elderly home. Residents themselves and the wheelchair surfaces might play an important role in facilitating the transmission, either by direct or indirect contacts, as contaminated surfaces have been reported to be an important transmission vector in some norovirus outbreaks [53,54]. It was also possible that this variable has served as an indicator for other risk factors, such as different characteristics of the residents; the health status or age structure, etc. might be different among homes with or without better wheelchair accessibility, which might contributed to different risk of norovirus transmission.

Partition between beds was found to be linked to reduced risk of norovirus outbreaks in Hong Kong elderly homes. Previous studies have showed that roommate exposure and neighborhood exposure are important risk factors for the transmission of diarrhea infection in health care facilities [36,37]. It was believed that the mechanisms of protective effects of partitions between beds might be related to that of these two exposures; to some extent, supply of partitions between beds could yield an immediate benefit, providing a physical barrier that limited the transfer of infection between the two areas [55]; and thus reduced the risks caused by roommate and neighborhood transmission.

There are a number of limitations to be considered when interpreting the results of the current study. The accuracy of the data obtained is of critical importance. Our outcome information was collected by an outbreak surveillance system; it was largely affected by the completeness of the reporting; under-reporting was a limitation of the current study. It was usually the larger outbreaks that received attention and were reported, and thus included in the study. The present study excluded eleven "self-care hostels", which might be one potential source of selection bias; however, when we included those homes in our analysis, which gave us a very similar results; so if the exclusion presented bias, it should not have been very serious. One should also be noted that it was an institutional-level study; some individual-level characteristics that might play an important role in the norovirus outbreaks were not taken into consideration [56], further investigations were needed to confirm the findings from the current study.

## Conclusions

In summary, this study evaluated the impact of institutional risk factors on norovirus outbreaks in Hong Kong elderly homes. Based on the present results, it is recommended that increased emphasis be placed on proper infection control measures, especially in homes with a larger capacity, a higher number of nursing staff, a higher staff-to-resident ratio, better wheelchair accessibility; partitions between beds should be considered as an effective infection control measure in elderly homes.

## Competing interests

The authors declare that they have no competing interests.

## Authors' contributions

LWT, SN, SCH and KL conceived and designed the study; HLL, SC, WMC and LWT performed the data management and data analysis; HLL, SCH, KL and LWT drafted the manuscript. All authors contributed to the revision of the manuscript and approved the submitted version of the manuscript.

## Pre-publication history

The pre-publication history for this paper can be accessed here:

http://www.biomedcentral.com/1471-2458/11/297/prepub
